# Home Exercise Interventions in Frail Older Adults

**DOI:** 10.1007/s13670-020-00326-6

**Published:** 2020-08-05

**Authors:** Alyssa D. Stookey, Leslie I. Katzel

**Affiliations:** 1 Department of Veterans Affairs and Veterans Affairs Medical Center, Geriatric Research, Education and Clinical Center (GRECC), Baltimore, MD, USA; 2 Division of Gerontology and Geriatric Medicine, Department of Medicine, University of Maryland School of Medicine, Baltimore, MD 21201, USA

**Keywords:** Frail, Older adults, Home exercise

## Abstract

**Purpose of Review:**

Frailty is characterized by decreased physiological reserve and increased risk of falls, disability, hospitalization, and mortality. Frail older adults may benefit from exercise interventions targeting their multiple problems and functional deficits; however, most research focuses on center-based interventions, which may present accessibility challenges for frail older adults. Therefore, the purpose of this review is to summarize the most recently published home-based exercise interventions for frail older adults living at home.

**Recent Findings:**

Eight manuscripts met inclusion criteria. Research interventions consisted of a variety of modes (strength, strength/nutrition, strength/flexibility/balance/endurance), duration (12 weeks to 6 months), frequency (2–7 days/week), and delivery methods (volunteer-led, videos on a tablet, manuals/brochures). Investigators examined the effects of home-based exercise on a variety of outcomes to include feasibility, frailty status, physical performance, lean body mass, skeletal muscle mass, other physiological outcomes, mental health, nutritional status, and incidence of falls in frail.

**Summary:**

This review demonstrates the feasibility and effectiveness of home-based exercise interventions to improve frailty, functional performance, nutritional status, and incidence of falls in frail older adults. However, the limited literature available provides conflicting reports regarding benefits for mental health outcomes and no evidence of a beneficial effect on skeletal muscle or lean mass. Future research is needed to shed light on the optimal components of home exercise programs most important for maximizing benefits for frail older adults, as well as the most effective delivery method.

## Introduction

Frailty, a geriatric syndrome characterized by decreased physiological reserve, impairs the ability to respond to stressors [1]. Frailty confers increased risk for falls, disability, hospitalization, and mortality [2–6]. The pathophysiology of frailty is multifactorial and not due to normal aging, but instead caused by age-related comorbid medical disease and other lifestyle, environmental, educational, and psychological risk factors [7]. There are two widely accepted approaches for defining and quantifying frailty; a phenotypic approach and a deficit-accumulation frailty index [8]. Even within these two broad approaches, numerous conceptual models and phenotypic criteria that employ a variety of assessment instruments have been proposed to define frailty [9]. Therefore, there is no “gold standard” for diagnosing frailty. The prevalence of frailty varies widely according to the criteria and instruments used, but the prevalence increases markedly with age, particularly in those > 80 years of age [10]. One of the most commonly cited phenotypic criteria for frailty are those developed by Fried et al. based on data from the Cardiovascular Health Study (CHS) [11]. It is important to recognize that frailty exists on a spectrum ranging from “prefrail” to failure to thrive, which represents severe end-stage frailty. Other approaches such as the deficit-accumulation frailty index focus on accumulation of multisystem health deficits, including comorbidities and disabilities [12, 13].

Frail older adults may benefit from interventions that target their multiple problems and functional deficits. Given the universal presence of weakness, low physical activity, and fatigue in those with frailty, exercise may be particularly beneficial for this population [14]. The majority of literature focuses on center-based exercise interventions, which may present accessibility challenges for older adults unwilling or unable to travel regularly outside of the home. Therefore, in this article, we will review recent home-based exercise interventions proposed to improve functional status and health in the frail elderly. In reviewing the literature, it is important to recognize that there is inconsistency in how “frailty” status is defined in these studies, which, in part, reflects the different conceptual models of frailty. Therefore, for this review, we broadly employ the term frailty to exam recently published home-based exercise interventions for frail older adults.

## Methods

**Fig. 1:**
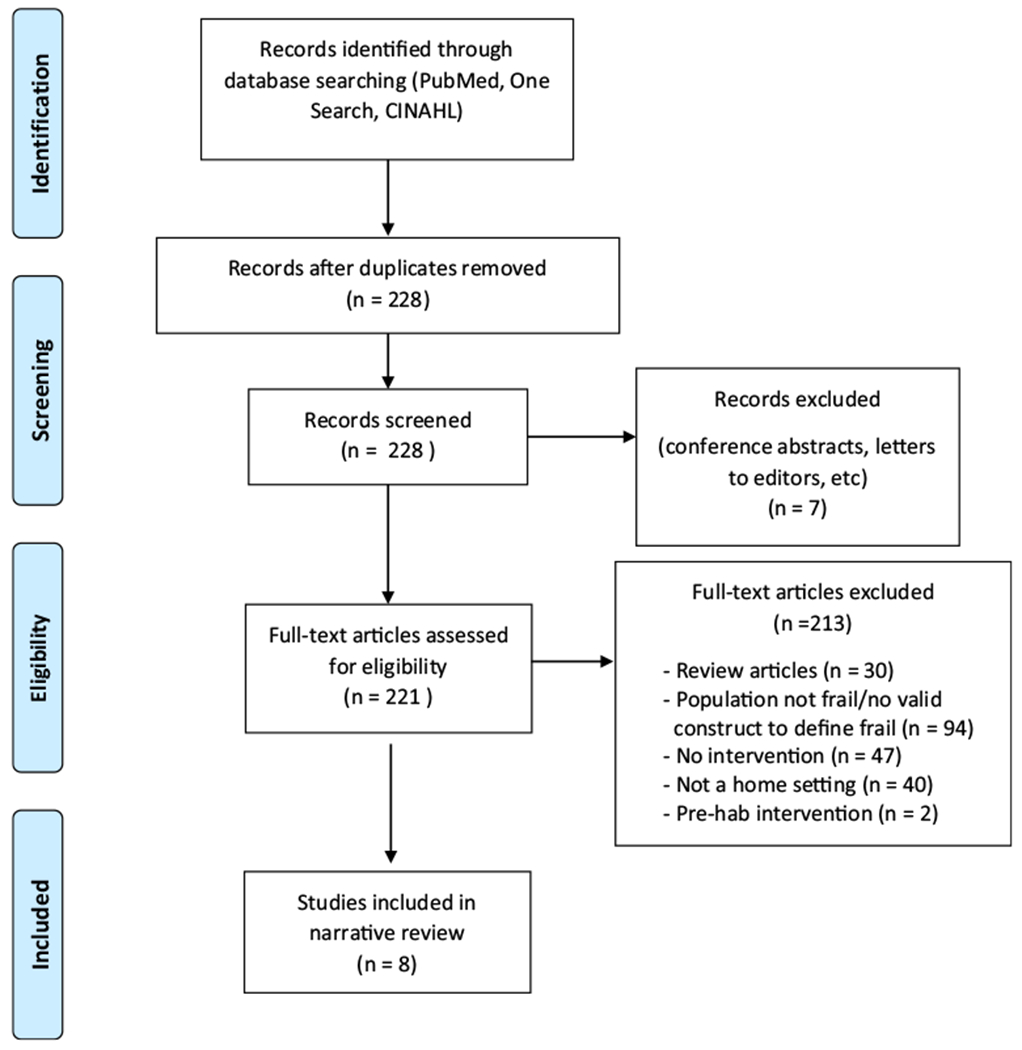
PRISMA flow diagram of study selection

Articles were identified through a search of One Search, which explores the University of Maryland Health Science and Human Sciences Library University of Maryland digital catalogue, eBook Collection (EBSCOhost), library collection, and > 100 databases, pubmed, and CINALHL. Search terms included frail+exercise+home, published in English covering the period of time from January 2014–December 2019. One Search uncovered 263 potential studies, while CINAHL and PubMed uncovered 105 potential studies, with considerable overlap (Fig. 1). Articles were screened and curated based on a review of abstracts and manuscripts. Full-text articles were assessed for eligibility. Only home-based interventions published within the last 5 years that examined the effects of home-based exercise in frail older adults living at home were included. Studies were excluded if subjects lived in an assisted-living facility/nursing home or if any of the exercise sessions took place outside the home. Studies that only reported the planned study design and those that only reported baseline data, review articles, or meta-analyses were also excluded. Due to the previously mentioned lack of a standard definition for frailty, only studies utilizing a valid construct to define frailty for inclusion criteria were included. In total, 8 studies were included in this narrative review [15–22] (results from one intervention was published in four separate articles) [15–18].

### Data Extraction

Data were extracted for the following variables: (1) study first author, (2) year and country of publication, (3) number of frail or pre-frail patients in sample, (4) a description of frailty definition, (5) inclusion criteria, (6) mean age of sample, (7) intervention, and (8) outcomes. Extracted data were independently verified (i.e., double verification) by both members of the study team.

## Results

### Participants and Study Characteristics

We reviewed 8 studies (Table 1) [15–22]. The analyzed interventions included a total sample of 1030 community-living older adults (53% women) with a mean age of 79.6 ± 7.1 years. Four studies were conducted in Austria [15–18], one in the UK [19], one in the Netherlands [20•], one in Japan [21], and the other in Taiwan [22•]. These studies used 5 different criteria for frailty: the Edmonton Frail Scale (EFS) [19], the Groningen Frailty Indictor (GFI) [20•]. Survey of Health, Ageing, and Retirement Group (SHARE-FI) [15–18], the Fried criteria (CHS) [22•], and the Kibon Checklist [21]. These studies examined the effects of exercise on a variety of outcomes such as feasibility, frailty status, physical performance measures, lean body mass, skeletal muscle mass, physiological outcomes, mental health, nutritional status, and incidence of falls in frail elderly. All outcome measures were collected pre- and post-intervention with no long-term follow-up. Two studies also performed mid-intervention outcome testing [20, 22].

### Interventions Characteristics

Among the included studies, type of exercise intervention varied to consist of strength training [19], combined strength and balance training [20•], combined strength training with nutrition education [15–18], and a multicomponent exercise program including strength, flexibility, balance, and endurance training [21, 22]. Additionally, the duration of the interventions ranged from 12 weeks to 6 months and frequency of exercise sessions varied from 2 to 7 times per week. Total volume of training ranged from 24 to 180 sessions over 12 weeks to 72–168 sessions over 6 months. Control groups to which interventions were compared consisted of social support and usual care. Delivery of the home-based exercise programs included buddy led sessions, instructional videos on a tablet, exercise DVD, and exercise manuals/brochures. Adherence was tracked a variety of ways to include weekly or specifically timed telephone calls and/or face-to-face visits, a necklace worn sensor, and self-report diaries.

### Effects of Home-Based Exercise Programs on Various Outcomes

#### Feasibility

Feasibility can be assessed in various ways to include retention rates, adherence/compliance to the intervention, and/or adverse events. Of the eight articles in this review, all three of these outcomes of feasibility were reported by two studies [19, 21], three reported retention rates and adverse events [16–18], one examined both retention and adherence [15], one study examined retention only [22•], and one study looked at adherence, as well as technical and operational feasibility [20•]. Completion rates for intervention groups (retention) ranged from 52.5–98%, which were determined by the number of participants who completed the entire interventional period and final outcomes testing [15–17, 19, 21]. Widespread adherence rates were reported ranging from 46–90% [15, 19, 20]. Interestingly, adherence was highest (90%) when 100% of exercise sessions were performed with an exercise “buddy” [15]. On the other hand, the lowest adherence rate (46%) was noted when only 3% of exercise sessions were led in-person by an instructor [19]. This suggests a certain level of accountability when home exercise is supervised in-person and may represent a critical component of successful home exercise interventions in frail, older adults. It is also important to note, the same study with the lowest adherence rate also required the highest training volume (3 times per day/5 days per week for the duration of the 12-week study), suggesting the possibility that training volume may also have an impact on adherence rates.

The most common adverse events reported were falls or hospital admissions. Clegg et al. [19] reported 7 of the 45 people in the intervention group (15.5%) and 8 of the 39 people in the control group (20.5%) had at least one fall. Furthermore, 2 participants in the exercise group (4.4%) and 4 participants in the control group (10.3%) had at least one hospitalization. The 4 adverse events reported by Haider et al. [16, 17] were not related to the intervention (2 deaths and 2 for medical reasons). Takatori et al. [21] reported no adverse events occurred and Kapan et al. [18] only had one adverse event (back pain). Frail older adults are at increased risk for falls, hospitalization, and death; therefore, these events, including the deaths, were anticipated.

Geraedts and colleagues [20•] focused solely on the feasibility of integrating a home-based strength and balance exercise program via tablet application for frail older adults. The dropout rate in this study was high with only 21 of 40 subjects completing the 6-month program. Of those who dropped out, 11 did so because of internet reception problems, 5 for medical reasons not related to the program, 2 due to illness of their spouse, and 1 subject died. Interestingly, 16 of the 19 dropouts (84%) occurred during the first 3 months. 60.9% of all participants (including non-completers) adhered to exercising with the tablet for the preferred 5 times per week while active in the study; however, 75.8% of completers were able to do so. Additionally, technical and operational feasibility showed a total of 249 incidents over the course of the 6-month study, which averages 0.8 incidents per week per participant. These incidents were categorized as technology-related (43.8 % of all issues; disconnection of tablet), connectivity-related (44.6%; internet issues), or participant-induced (11.6%; accidental removal of application from home screen and opening too many screens). Feasibility, as related to participant satisfaction, demonstrated completers (*n* = 21) reported an average satisfaction score of 4.2 ± 0.2 (range 0–5 with higher scores representing greater satisfaction) and mean satisfaction score reported by dropouts (*n* = 11) was 2.0 ± 0.9. This study demonstrates the promise for the integration of technology as feasible for exercise delivery in frail elderly. Unfortunately, this study did not provide information on functional status of subjects at baseline or post-intervention even though such testing was included in the manuscript describing their study protocol [23].

#### Frailty Status

Two studies examined the effects of home-based exercise interventions on frailty status [12, 22]. One study defined frailty based on SHARE-FI score [15], while the other utilized the CHS criteria for frailty [22•]. Both studies included pre-frail and frail adults ≥ 65 years of age. Luger and colleagues [15] randomly assigned participants to either a combined resistance training and nutrition intervention (PTN) or a social support (So Su) group (control) and demonstrated a significant reduction in frailty score for both groups (− 17% PTN and − 16% SoSu). However, there was no significant difference between groups. Hsieh et al. [22•] randomly assigned 319 participants to one of four groups, exercise only, nutrition only, combined exercise and nutrition, or usual care (control), who were followed up during a 3-month intervention period and 3-month self-maintenance period. The exercise protocol included resistance, flexibility, balance, and aerobic training. Results showed a significant reduction in frailty for all three interventional groups compared with controls. However, participants in the combined exercise/nutrition group had greater improvements in frailty scores at the end of the 6-month intervention (− 0.34; 95% CI: − 0.52 to − 0.16; *p* < 0.001) as compared with either the exercise only (− 0.23; 95% CI: − 0.41 to − 0.05; *p* = 0.012) or nutrition only group (− 0.28; 95% CI: − 0.46 to − 0.11; *p* = 0.002). Both studies demonstrated significant reductions in frailty demonstrating the importance of incorporating physical activity, as well as nutrition education into the lives of older frail adults.

#### Physical Performance

Four studies examined the effects of home-based exercise on physical performance outcomes including handgrip strength, timed up and go (TUG), short physical performance battery (SPPB) [24], gait speed, and balance [16–19, 21, 22].

##### Handgrip Strength

Two studies assessed handgrip strength, which can be indicative of overall upper body strength [15, 22]. Hsieh and colleagues [22•] found significant improvements in handgrip strength for all three intervention groups as compared with controls. The largest improvements (change from baseline) were seen in the exercise only group (+ 2.00 kg; 95% CI: 1.16 to 2.84; *p* < 0.001), followed by the combined exercise and nutrition group (+ 1.30 kg; 95% CI: 0.45 to 2.14; *p* = 0.003) and, finally, the nutrition only group (+ 1.09 kg; 95% CI: 0.26 to 1.93; *p* = 0.011). The other study to analyze handgrip strength [16•] was the same intervention described above by Luger et al. [15]. In the PTN group, handgrip strength significantly improved by 2.4 kg, whereas no improvements were seen in the control group (SoSu). Furthermore, results demonstrated that frail participants were more likely to improve their handgrip strength as compared with their pre-frail counterparts.

##### Function and Mobility

Four studies assessed function and mobility [16–19, 21, 22]. Clegg and colleagues [19] examined the effects of a 12-week home-based strength and mobility program versus usual care (control) on mobility in frail older adults. Mobility was assessed by the TUG test [25]. Although the results demonstrated TUG times increased in both groups (52 to 62.4 s in the intervention group and 57.9 to 97 s in control—an indication of mobility deterioration), frail older adults in the exercise group experienced less reduction in mobility (10.4 s) when compared with controls (39.1 s). Haider et al. [16•] assessed mobility with the SBBP, which incorporates aspects of balance, gait speed, and lower extremity strength. Results demonstrated a significant improvement in SBBP scores for both the PTN (mean change 1.2; 95% CI: 0.3 to 2.1; *p* = 0.009) and SoSu (0.5; 95% CI: 0.1 to 0.9; *p* = 0.011) groups. Significant improvements in the PTN group were attributed to significant changes in balance (0.4; 95% CI: 0.0 to 0.8, *p* < 0.001) and lower limb strength (chair sit-to-stands; 0.6; 95% CI: 0.2 to 1.0, *p* = 0.003), whereas improvements in the SoSu group were due to significant improvements in balance (0.5; 95% CI: 0.2 to 0.8, *p* = 0.002). There were no significant differences in gait speed for either group. Hsieh et al. [22•] also examined the effects of their individualized home exercise/nutrition intervention on gait speed (10-m walk). When compared with the control group. all three intervention groups (exercise, nutrition, and combined exercise/nutrition) had significant improvements in gait speed, with greatest improvements seen in the nutrition (− 0.81 s; 95% CI: − 1.37 to − 0.25, *p* < 0.001) and combined exercise/nutrition groups (− 0.81 s; 95% CI: − 1.38 to − 0.24, p < 0.001). Takatori and colleagues [21] assessed function by testing gait speed, TUG, and balance. When compared with controls, the intervention group had greater improvements in TUG time (− 0.15 ± 1.21 vs 0.24 ± 1.07 s, *p* = 0.022), sit-and-reach (0.6 ± 5.8 vs 2.8 ± 6.8, *p* 0.046), and postural sway with eyes open (0.3 ± 0.47 vs − 0.15 ± 0.70, *p* = 0.052). No significant differences were reported for gait speed, postural sway with eyes closed, or functional reach test.

##### Muscle Strength and Flexibility

Two studies investigated the effects of home exercise on lower body muscle strength and only one study on upper and lower body flexibility in frail elderly [21, 22]. When compared with controls, Hsieh and colleagues [22•] reported the exercise and combined exercise/nutrition groups had significant improvements in lower extremity strength (mean difference 2.95; 95% CI: 1.99 to 3.90, *p* < 0.017 and 4.05; 95% CI: 3.11 to 5.00, *p* < 0.017, respectively), as well as upper (5.21; 95% CI: − 3.10 to 7.31, *p* < 0.017 and 4.29; 95% CI: 2.81 to 7.03, *p* < 0.017, respectively) and lower body flexibility (2.33; 95% CI: 0.96 to 3.70, *p* < 0.017 and 2.17; 95% CI: 2.17 to 3.55, *p* < 0.017, respectively), whereas no significant differences were noted for these outcomes in the nutrition only group. Takatori et al. [21] assessed lower extremity strength (30-s sit-to-stand test and knee extension with a hand-held dynamometer). When compared with controls, the intervention group had significant improvements in 30-s chair stands (− 0.3 ± 4.3 vs 2.00 ± 3.8, *p* = 0.007) and knee extensor strength (− 0.20 ± 8.8 vs 2.61 ± 9.9, *p* = 0.035).

#### Lean Mass and Muscle Mass

One study analyzed the effects of home exercise/nutrition on lean body mass and skeletal muscle mass in frail older adults [16•]. Both lean body mass and muscle mass were assessed with bioelectrical impedance analysis. No significant within-group differences were found in either the PTN or SoSu group for lean body mass (mean difference 0.4, *p* = 0.55 and 0.5, *p* = 0.24, respectively) or skeletal muscle mass (0.3, *p* = 0.20 and 0.2, *p* = 0.18, respectively). Furthermore, no significant within-group differences were found for lean body mass (− 0.04, *p* = 0.61) or skeletal muscle mass (0.1, *p* = 0.814)

#### Other Physiological Outcomes

Two studies examined other variable related to physiological outcomes (voluntary peak cough flow (VPCF), lip closure force (LCF) [21], and inflammatory parameters [17]). In Japan, there has been an increase in mortality rate due to pneumonia in frail older adults [26]. As a result, Takatori et al. [21] examined the effect of stretching, balance, and lower limb strength exercises on VPCF and LCF (both important in reducing risk of aspiration pneumonia) in older, frail women. Results demonstrated that the intervention group had significant improvements in VPCF, but not LCF when compared with controls (7.4 ± 73.7 vs 42.7 ± 95.4, *p* = 0.004 and 0.07 ± 3.11 vs − 0.09 ± 4.09, *p* = 0.424, respectively).

Haider and colleagues [17] examined the effect of PTN versus usual care control on inflammatory parameters in frail older adults. Increased inflammatory parameters have been linked to frailty [24, 27]. This study examined inflammatory parameters including tumor necrosis factor alpha (TNF-*α*), interleukin 6 (IL-6), C-reactive protein (CRP), and total leukocyte count. Following the 12-week intervention, only a significant difference was reported between PTN (mean change 0.08; 95% CI: − 0.16 to 0.32) and controls (0.46; 95% CI: 0.07 to 0.85) for CRP (*p* = 0.040); however, IL-6 and CRP remained the same in the PTN group, but increased in the control group (SoSu). No changes in TNF-*α* or total leukocyte count were reported for either group. Participants with greater improvement in physical performance were more likely for inflammatory markers to decrease or remain the same. This demonstrates the ability for an exercise and nutrition program to potentially delay an increase in inflammation parameters in frail elderly.

#### Falls

Interestingly, only one study assessed fall rate [21]. Falls were monitored for the duration of the 6-month interventional period. Results demonstrated that a 6-month exercise program significantly reduced the incident of falls in the exercise group (26% reported falls pre-intervention and only 12% reported a fall post-intervention; *χ*^2^ = 8.20, *p* < 0.0), whereas there were no significant reductions in the control group (32% pre- and 22% post-intervention, *χ*^2^ = 3.09, *p* = 0.07). Additionally, the difference in number of falls between groups was significant (control = *χ*^2^ = 5.38, *p* < 0.05).

#### Mental Health: Quality of Life and Depression

Three studies examined the effects of home exercise interventions on mental health outcomes in frail older adults [18, 19, 22]. Two studies assessed depression; both utilized the Geriatric Depression Scale. Neither found a significant difference in depression when compared with controls. [19, 22] Clegg and colleagues [19] demonstrated a non-significant between-group difference for depression (0.2; 95% CI: 1.1 to 1.5) and Hseih et al. [22•] found the same for the exercise (− 0.43; 95% CI − 0.86 to 0.01), nutrition (− 0.42; 95% CI − 0.85 to 0.01), and combination exercise/nutrition groups (− 0.38; 95% CI − 0.82 to 0.06) when compared with controls. Quality of life (QOL) was examined with the 12-item Short Form Health Survey in one study [22•], the EuroQol Group 5-Dimension Self-Report Questionnaire in one study [19], and the World Health Organization Quality of Life Instrument (WHOQOL-BREF and -OLD) in the other [18]. When compared with controls at 6 months, Hsieh and colleagues [22•] found no significant difference in health-related QOL in the exercise or combination exercise/nutrition groups; however, they did report a significant difference in the nutrition group (mean change 2.12; 95% CI: 0.49–3.75; *p* < 0.017). Clegg et al. [19] found no significant difference in QOL at the end of their 12-week exercise intervention between exercise and control groups (between-group difference 0.04; 95% CI: − 0.09 to 0.18). After the 12-week study, Kapan and colleagues [18] found no significant differences between the exercise group and controls for any QOL domains, expect for past, present, and future activities. For this domain, the PTN group had significant improvements when compared with controls (mean change 3.66; 95% CI 0.13 to 7.18; *p* = 0.039). However, significant within-group differences for overall QOL (mean change 5.6; 95% CI 0.95 to 10.33; *p* < 0.05), social relationship (4.5; 95% CI 0.38 to 8.59; *p* < 0.05), and social participation (3.8; 95% CI: 0.12 to 7.57, *p* < 0.05) were reported for the PTN group. No within-group differences were noted in the control group. Significant improvements in physical domains of QOL in the PTN group could be explained by their more active lifestyle.

#### Nutrition Status

Two studies assessed nutritional status in frail older adults [15, 22]. Both of these studies had a nutrition component built into their intervention. Luger and colleagues [15] utilized the Mini Nutritional Assessment Long Form (MNA-LF), a validated nutrition instrument for adults ≥ 65 years [28], to assess nutrition status pre- and post-intervention, whereas Hsieh et al. [22•] tracked nutrition intake through dietary recall during dietary consultations. Results demonstrated a significant increase in MNA-LF score as compared with just social support alone (control group) [15]. Furthermore, those at a higher risk for malnutrition pre-intervention were reported to have the largest improvements in nutritional status. Significant improvements in nutritional status were primarily associated with fluid, as well as fruit and vegetable intake. Hsieh and colleagues [22•] did not find any significant differences in nutritional outcomes between the exercise or nutrition groups; however, the nutrition intervention group did increase intake of total calories, protein, carbohydrates and fat at all timepoints (1, 3, and 6 months).

## Discussion

The majority of aging literature supports the beneficial effects of multicomponent exercise interventions to prevent functional decline, reduce levels of disability, and improve mental health in older adults. However, the center-based nature of this research may present a challenge for frail older adults due to the travel requirement. Therefore, home-based exercise interventions may be a feasible alternative for this population by eliminating the challenge of having to leave their home. This review provided a summary of results from recent research examining the impact of home-based exercise interventions on various outcomes in community-living frail older adults. There are relatively few randomized control trials that are truly “home”-based interventions in this population, with most studies claiming to be as such, but the exercise interventions are actually conducted in nursing homes or assisted-living facilities. The results of this review demonstrate the feasibility and effectiveness of home-based exercise interventions to improve frailty, functional performance, nutritional status, and incidence of falls in frail older adults living at home. However, the limited literature available provides conflicting reports regarding the effects of home-based exercise on mental health outcomes. Furthermore, there is a lack of evidence for a beneficial effect of home exercise on muscle/lean mass in older, frail adults.

When interpreting the evidence of the presented frailty research, it is important to do so with caution due to certain challenges. More specifically, the lack of a gold standard definition for frailty makes it difficult to compare results of investigations given the numerous criteria used to determine “frail.” This lack of consistency with regard to defining frailty could explain why only two studies focused on frailty as an outcome measure. Determining a valid and reliable gold standard criterion for measuring frailty would allow for a better analysis of the effects of home exercise interventions on this specific outcome and make it more feasible to compare results across various investigations.

Furthermore, the most effective and beneficial type of exercise intervention for frail older adults is not well understood. Utilization of different modes of exercise and duration of interventions as well as varying exercise frequency, intensity, and duration of exercise makes it difficult to determine which components are most important to maximize benefits of home exercise in frail older adults. Additionally, the delivery of home-based exercise interventions lacks consistency as well. Some studies employ trained exercise buddies to deliver the exercise programs, while others use instructional exercise manuals, videos on tablets, and/or manuals/brochures. The manner in which exercises were delivered to participants may explain the wide range of adherence rates noted in this review. Evidence supports higher adherence rates for exercise programs that are supervised [29]. This review supports this notion as adherence rates were highest when training “buddies” administered all exercise sessions with the participants [15]; whereas adherence rates were lowest when participants used an exercise manual to perform the exercises with sporadic in-person contact with physiotherapists [19]. Supervision may add a level of accountability not achieved with sporadic supervision or none at all, thus, leading to higher adherence rates. Differences in training volume may also impact adherence. Not only were lower adherence rates noted based on a lack of in-person supervision but that same intervention also had the highest volume of training. The appropriate level of in-person supervision as well as proper training volume is not well understood, but evidence sheds light on the potential impact these two components may have on adherence, which in turn impacts the effectiveness of the interventions.

To our knowledge, there are no home-based exercise trials in frail older adults that incorporate long-term follow-up of important outcomes. More research is needed to determine whether significant improvements are maintained post-intervention and if frail older adults continue to exercise after the study ends. This could provide insight into factors that may influence long-term adoption of an active lifestyle, as well as whether frail older adults can continue to see improvements in various outcomes important for function, quality of life, and overall wellness.

Several limitations are worth noting. First, given the variability in how frail subject populations are described and defined, it is possible that our literature search did not include all of the relevant recent home-based interventions in older adults with frailty. Second, this review was not intended as a meta-analysis, but rather as a critical review of recent home-based exercise interventions in frail older individuals. Third, we focused on interventions conducted at home in community-dwelling older adults and did not review interventions in nursing home patients. Finally, many exercise interventions are in older individuals described as being pre-frail, and we have not included these studies in our review as we focused on individuals with frailty.

## Conclusion

While additional research is needed, the literature suggests that home-based exercise is feasible and beneficial for frail, older adults living at home. However, to expand on the knowledge base related to home exercise in frail elderly, future research is needed to shed light on the optimal components (frequency, duration, intensity, mode) of home exercise necessary to provide the most benefits to frail older adults and whether benefits are sustainable. Additionally, future research should also focus on determining the most effective delivery method of exercise in the home for frail elderly and on the contributions of nutrition counseling, social support, and occupational therapy to improve independence and cognitive function as part of a multimodal intervention. Finally, more research is needed to examine long-term follow-up of important outcomes and to identify the underlying mechanism(s) responsible for positive effects of exercise on various outcomes in frail older adults.

## Figures and Tables

**Table 1:** Overview of home-based exercise research in frail older adults

Authors	Subjects	Inclusion criteria	Intervention	Outcomes
Clegg et al. [19] (2014)	*N* = 84 Men and women Ave age: 78.7 + 9.2 years Location: UK	1. Living at home 2. Housebound (unable to leave house W/o assistance of another person 3. Spectrum of frailty > 30 s = level I (simple chair ex) 20–29 s = level 2 intermediate level < 20 s = level 3 independently mobile 4. Edmonton Frail Scale (EFS) (> 8 = frail w/ max score of 17 = highest level of frailty)	Duration: 12 weeks Groups: 1. Physical activity (*n* = 45) 2. Control group: usual care form primary healthcare team (*n* = 39) Physical activity intervention details: Frequency: 3 times per day/5 days per week Mode: strengthening exercises Progression: 5 reps to 10 then 15 Delivery: exercise manual with 5 face-to-face home visits & 7 phone calls by trained physiotherapists)	Timed up and go Barthel Index of ADL (assess function/ADL) QOL (EQ-D5) Depression—Geriatric Depression Scale
Luger et al. [15] (2016)	*N* = 80 Men and women Ave age: 82.8 + 8.0 years Location: Austria	1. > 65 years 2. Live in own homes 3. Pre-frail or frail based on frailty instrument of the Survey of Health, Ageing, and Retirement Group (SHARE-FI) 4. Able to walk with or without walking aid	Duration: 12 weeks Groups: 1. Physical activity and nutrition program (PTN) (*n* = 39) 2. Control group: Social support (SoSu) (*n* = 41) PTN group: Exercise details: Frequency: 2 times per week (plus encouraged 1×/week. on own) Mode: Strength exercises 2 sets of 6 strength exercises, discussed nutritional topics, social support Progression: 2 × 15 until failure—if all reps could not be completed reps increased 1st then intensity progressed (stronger band) Each session: Warm up with mobilization, circuit of exercises (mini squats in front of a chair, chest press (res band), ab exerc, hip extensions standing, reverse flys and shoulder press with band) Delivery: Trained lay volunteers (“Buddies”) for all sessions Nutrition details: Focused on fluid, protein, and energy intake Control details: SoSu only	Nutrition (Mini Nutritional Assessment Long Form) Frailty status (SHARE-FI)
Takatori et al. [21] (2016)	*N* = 266 Women only Ave age: 75 + 5.0 yrs Location: Japan	1. Frail elderly based on Kibon Checklist 2. No dementia diagnosis	Duration: 6 months Groups: 1. Exercise (*n* = 148) 2. Control (*n* = 118) Exercise details: Frequency: At least 3 times per week Mode: Stretching, breathing, balance, and strength exercises Progression: No details provided Each session: 5-min long Delivery: One session with physical therapist to learn exercises Then self-lead with brochure and DVD of exercise program Control details: Advised to maintain or improve daily activity and attended lectures on health Duration: 6 months Group: 1. Physical activity Physical activity Intervention details: Frequency: 5 times per week Mode: Strength and balance exercises Progression: 10 min/day and progressed to 45 min/day Delivery: Tablet for exercise instruction and necklace worn for daily activity registration 1st 3 months: Supervised via weekly telephone for coaching 2nd 3 months: Not contacted at all, but could call coach if needed necklace worn sensory for daily activity registration Duration: 12 weeks Groups: 1. Physical activity and nutrition program (PTN) (*n* = 39) 2. Control group: Social support (SoSu) (*n* = 41) PTN group: Exercise details: Frequency: 2 times per week (plus encouraged 1×/week. on own) Mode: Strength exercises 2 sets of 6 strength exercises, discussed nutritional topics, social Progression: 2 × 15 until failure—if all reps could not be completed reps increased 1st then intensity progressed (stronger band) Each session: Warm up with mobilization, circuit of exercises (mini squats in front of a chair, chest press (res band), ab exerc, hip extensions standing, reverse flys and shoulder press with band) Delivery: Trained lay volunteers (“Buddies”) for all sessions Nutrition details: Focused on fluid, protein, and energy intake Control details: SoSu only	Swallow-related function: Voluntary peak cough flow (VPCF) Lip closure force Balance (static and dynamic) Static: Stabilometer with open and closed eyes Dynamic: 5 m walk, timed up and go, functional Reach test Lower limb strength (dynamometer, 30-s chair stands) Flexibility (sit-and-reach Test) Feasibility Adherence Retention Feasibility Adherence Technical and operational feasibility Determinants of participation Participant satisfaction Hand grip (dynamometer) Short physical performance battery Muscle mass (BIA) Dropout rate Adverse events Adherence freq/duration home visits # sets per home visit p#p oofr texercises done per visit # reps # of circuits completed between visits
Haider et al [17] (2017)	*N* = 53 Men and women Ave age: 82.4 + 8.2 years Location: Austria	1. > 65 years 2. Live in own homes 3. Pre-frail or frail based on SHARE-FI 4. Able to walk with or without walking aid	Duration: 12 weeks Groups: 1. Physical activity and nutrition program (PTN) (*n* = 35) 2. Control group: Social support (SoSu) (*n* = 23) PTN group: Exercise details: Frequency: 2 times per week (plus encouraged 1×/week. on own) Mode: Strength exercises 2 sets of 6 strength exercises, discussed nutritional topics, social support Progression: 2 × 15 until failure—if all reps could not be completed reps increased 1st then intensity progressed (stronger band) Each session: Warm up with mobilization, circuit of exercises (mini squats in front of a chair, chest press (res band), ab exerc, hip extensions standing, reverse flys and shoulder press with band) Delivery: Trained lay volunteers (“Buddies”) for all sessions Nutrition details: Focused on fluid, protein, and energy intake Control details: SoSu only	
Kapan et al. [18] (2017)	*N* = 80 Men and women Ave age: 82.6 + 8.1 years Location: Austria	1. > 65 yrs 2. Live in own homes 3. Pre-frail or frail based on SHARE-FI 4. Able to walk with or without walking aid	Duration: 12 weeks Groups: 1. Physical activity and nutrition program (PTN) (*n* = 39) 2. Control group: Social support (SoSu) (*n* = 41) PTN group: Exercise details: Frequency: 2 times per week (plus encouraged 1×/week. on own) Mode: Strength exercises 2 sets of 6 strength exercises, discussed nutritional topics, social support Progression: 2 × 15 until failure—if all reps could not be completed reps increased 1st then intensity progressed (stronger band) Each session: Warm up with mobilization, circuit of exercises (mini squats in front of a chair, chest press (res band), ab exerc, hip extensions standing, reverse flys and shoulder press with band) Delivery: Trained lay volunteers (“Buddies”) for all sessions Nutrition details: Focused on fluid, protein, and energy intake Control details: SoSu only	Quality of life WHOQOL-OLD and WHOQOL-BREF)
Hsieh et al. [22] (2019)	*N* = 319 Men and women Ave age: 71.6 + 5.7 years Location: Taiwan	1. > 65 years 2. Cardiovascular Health Study criteria for frail and pre-frail (Fried) 3. Walk 14 m independently 4. Living in the home Exercise intervention details: Frequency: 3–7 sessions per week Mode: Strength, flexibility, balance, and endurance training Progression: 5 min to 60 min/session (repetitions based on participant’s capabilities) Delivery: Self-lead Nutrition intervention details: Weight maintenance with adequate food intake achieved with a designated number of servings of 6 major food groups (dairy, protein, vegetables, fruits, nuts, seeds, plant oils, grains/roots) Control group details: Regular medical care with telephone contact	Duration: 6 months (3-month intervention + 3-month follow-up) Groups: 1. Exercise only—individualized 2. Nutrition only 3. Exercise plus nutrition (combo) 4. Control group	Frailty (Primary; CHS) Physical performance (10 m, flex, lower ST, balance, handgrip, PA vol) Depression (Geriatric Depression Scale) Quality of life (Short Form Health Survey)
